# A refined SMOTE-ENN optimization method based on machine learning for heart rate variability data classification

**DOI:** 10.3389/fdgth.2026.1749570

**Published:** 2026-02-12

**Authors:** Biao Zhang, Muzi Liang, Yuanlun Zhou, Binbin Ji, Meng Han, Hongyan Li, Xufeng Lang, Yihua Song, Run Gao, Zuojian Zhou, Xuebin Qiao

**Affiliations:** 1 School of Artificial Intelligence and Information Technology, Nanjing University of Chinese Medicine, Nanjing, China; 2 Jiangsu Province Engineering Research Center of TCM Intelligence Health Service, Nanjing University of Chinese Medicine, Nanjing, China; 3 The Affiliated Brain Hospital of Nanjing Medical University, Nanjing, China

**Keywords:** depression detection, heart rate variability, imbalanced data, machine learning, over-sampling technique

## Abstract

**Introduction:**

The classification of imbalanced heart rate variability (HRV) data utilizing machine learning algorithms is of considerable significance for the early detection of depression. In this work, a refined SMOTE-ENN hybrid optimization method based on machine learning algorithms is proposed, which achieves precise classification of Autonomic Nervous System (ANS) states using imbalanced and limited HRV data.

**Methods:**

The refined Synthetic Minority Over-sampling Technique generates new minority class samples through in-line and off-line linear interpolation. Subsequently, the refined Edited Nearest Neighbor under-sampling algorithm is employed to remove most of the noisy data while retaining selected boundary data to reduce the overfitting risk. Four machine learning algorithms are employed to classify the optimized HRV data using the refined SMOTE-ENN method from 321 participants, including support vector machine, random forest, neural network, and K-nearest neighbors.

**Results:**

The results indicate that the classification accuracy of all four methods surpasses 91%, with the AUC (Area Under the Curve) values exceeding 0.92 following the refined SMOTE-ENN optimization. In comparison to the classification results with classical SMOTE optimization of the four machine learning algorithms, the mean accuracy, precision, recall, and F1 score improved by 0.12, 0.12, 0.10 and 0.11, respectively. Feature importance analysis reveals that SDNN (standard deviation of NN intervals) has the most significant impact on HRV classification results, reflecting its influence on ANS.

**Discussion:**

The refined SMOTE-ENN optimization method enhances the detection performance of the machine learning algorithms for classifying imbalanced HRV data, providing valuable technical support for the early detection of depression.

## 1 Introduction

Depression can be seriously harmful to one's physical and mental health (1, 2). Early detection of depression is crucial for timely intervention and for improving patient prognosis. Currently, the commonly used methods for early detection of depression are health questionnaires (3), sleep pattern analysis (4), and heart rate variability (HRV) analysis (5). Health questionnaires, such as the Patient Health Questionnaire (PHQ-9) and General Anxiety Disorder Scale (GAD-7), are widely used due to their simplicity and convenience (6). However, self-reported data are often subjective and susceptible to biases, such as patients' preconceptions and memory recall. To reduce the subjectivity inherent in health questionnaires, sleep pattern analysis has been proposed as an alternative method for assessing mental health status (7). Nevertheless, sleep issues may arise from multiple causes, such as physical illness, lifestyle, and even the sleep environment. Additionally, the accuracy of sleep tracking by consumer wearable devices remains limited, so the reliability of sleep pattern analysis needs further improvement (8).

In recent years, HRV has gained increasing attention as an objective and quantifiable method for early depression detection (9). Compared with health questionnaires and sleep pattern analysis, HRV has the advantages of objectivity and easy quantification, so it has been widely applied in depression detection. HRV refers to the variability of the time interval between consecutive heartbeats and is an important indicator of autonomic nervous system (ANS) function (10). It reflects the balance between the sympathetic nervous system (SNS) and the parasympathetic nervous system (PNS). Lower HRV is usually associated with increased SNS activity and decreased PNS activity (9). In 2019, Hartmann et al. (11) studied the HRV between 62 depressive individuals without antidepressant medication and 65 healthy controls. The results revealed differences in HRV parameters between the depressive patients and the healthy controls at baseline, and the changes in HRV parameter values are correlated with changes in symptom severity of depression. In 2018, Koch et al. (12) conducted a comprehensive analysis of 21 studies on patients with major depression, including 2,250 depressive patients and 1,982 healthy controls. The results showed that all the measured HRV parameters of patients with major depression were lower than those of healthy controls. In 2023, Wu et al. (13) conducted a meta-analysis of 43 papers on depressive patients. The comprehensive analysis results showed that the HRV measurement parameters of depressed individuals were lower than those of healthy controls, except for LF/HF, suggesting that depressed people may be at a higher risk of cardiovascular diseases than healthy people.

Although HRV is closely related to depressive symptoms and can be used as an effective biological indicator for early detection of depression, imbalanced data usually exist when collecting HRV data from participants, especially when sample sizes are small (14). Imbalanced data indicates that the sample size of the majority class is much larger than the sample size of the minority class, and it is quite common in medical diagnosis (15). When the value of minority class samples overwhelms that of majority class samples, the effect of imbalanced data becomes particularly significant, such as in the detection of malignant tumors (16). However, traditional classification algorithms usually assume that the training dataset is balanced, or assume that the misclassification loss of different classes is the same (17, 18), which is not applicable in imbalanced diagnosis data. Consequently, how to train machine learning algorithms that can effectively classify imbalanced HRV data remains an urgent problem (19).

To address this issue, data resampling techniques have been widely adopted to adjust the class distribution. Among them, the Synthetic Minority Oversampling Technique (SMOTE) and its variants have proven particularly effective in medical signal analysis. For instance, Hussain et al. (20) demonstrated that applying SMOTE to multimodal HRV features significantly improved the sensitivity and overall accuracy of congestive heart failure detection, verifying that synthetic oversampling can preserve the discriminative power of physiological signals. Beyond standard oversampling, hybrid strategies have also been proposed to further refine classification boundaries. Xu et al. (15) introduced a method combining SMOTE with Edited Nearest Neighbor (ENN), which not only balances the class distribution but also eliminates noisy samples from the majority class. Their results across various datasets suggest that such hybrid resampling is superior in handling the noise and overlap often found in complex medical data. These studies collectively indicate that data resampling is a viable strategy for improving the classification performance of imbalanced physiological data. Currently, studies on imbalanced data based on SMOTE typically use original datasets with large sample sizes (20–22), generally ranging from several thousand to millions. However, its effectiveness in the specific context of depression detection using limited and sparse HRV samples remains to be fully explored.

In this work, a refined SMOTE-ENN hybrid optimization method based on machine learning algorithms is proposed to address the classification challenges of limited and imbalanced sample distribution. The refined Synthetic Minority Over-sampling Technique (r-SMOTE) generates new minority class samples by in-line and off-line linear interpolation. Afterwards, the refined Edited Nearest Neighbor (r-ENN) under-sampling method is employed to remove most of the noisy data and retain selected boundary data to reduce the overfitting risk. The performance of the refined SMOTE-ENN method is demonstrated by optimizing the imbalanced HRV data from 321 participants, with each record containing nine features. These data are divided into seven ANS states, with minority class samples comprising less than 5% of the total data.

Four machine learning algorithms are employed to evaluate the classification performance after data optimization, including Support Vector Machine (SVM), Random Forest (RF), Neural Network (NN), and K-Nearest Neighbor (KNN). The classification results show that following refined SMOTE-ENN optimization, the overall classification accuracy of the four machine learning algorithms exceeds 91%, with the AUC (area under the receiver operating characteristic curve) values surpassing 0.92. In comparison to the classification results with classical SMOTE optimization of the four machine learning algorithms, the mean accuracy, precision, recall, and F1 score improved by 0.12, 0.12, 0.10 and 0.11, respectively. The refined SMOTE-ENN method improves the detection performance of machine learning algorithms in classifying limited and imbalanced data, providing valuable technical support for the early detection of depression.

Currently, most public datasets focus on binary classification, e.g., Stress vs. Rest in WESAD, or Arrhythmia vs. Normal in MIT-BIH. To the best of our knowledge, there is no public dataset that provides the fine-grained, 7-category taxonomy of autonomic nervous system states used in this study. This scarcity actually highlights the novelty and clinical value of the proposed categorization system. On the other hand, classical SMOTE positions newly generated samples on the lines connecting the original samples in the sample space. The refined SMOTE-ENN introduces off-line interpolation, which expands the data space and increases the diversity of generated data through nonlinear transformations introduced by trigonometric functions, overcoming the limitations of linear interpolation in the traditional SMOTE method. The proposed method demonstrates good optimization capabilities for imbalanced datasets, especially when the dataset size is small.

## 2 Materials and methods

### 2.1 Samples and data

**Figure 1:**
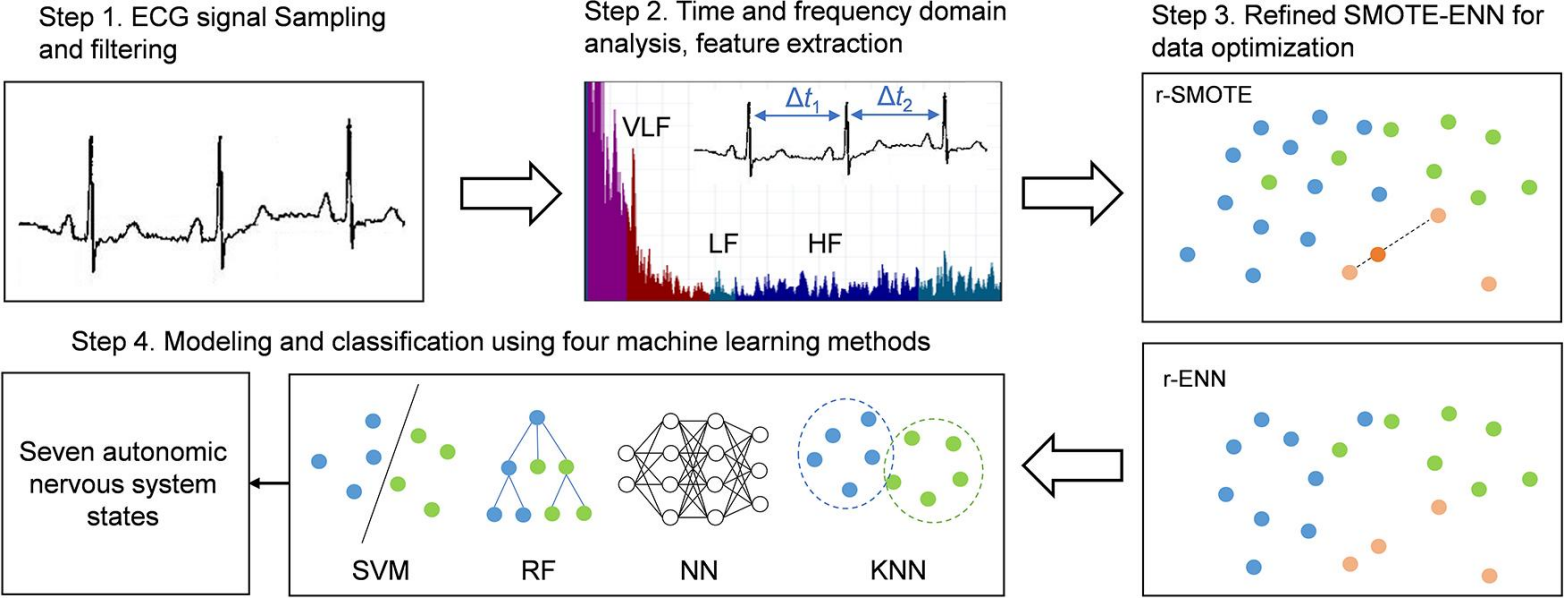
Overall flowchart of the experiment. The participants' ECG signals are collected using the 6-lead method, and nine HRV parameters are calculated. The refined SMOTE-ENN method is applied to optimize the imbalanced HRV data, followed by the use of four machine learning algorithms to classify the optimized data.

The samples were recruited from 321 participants (89 males and 232 females) between July and September of 2024 in the Affiliated Brain Hospital of Nanjing Medical University, Nanjing. The mean age is 33.16 ± 15.41 years (mean ± SD). The mean height, weight, and Body Mass Index (BMI) are 165.23 ± 8.02 cm, 62.52 ± 13.44 kg, and 22.78 ± 3.89 kg/m^2^, respectively. The experimental flowchart is shown in Figure 1. The participants' electrocardiogram (ECG) signals are filtered to remove baseline drift, myoelectric interference, and 50 Hz power frequency noise. The filtered signals are then digitally recorded using a high-speed, high-precision analog-to-digital converter, and the participants' information is anonymized.

Frequency and time domain analysis methods are employed to calculate nine HRV parameters based on the ECG signals, which are used as the features of the training dataset, as shown in Table 1. These nine parameters possess strong scientific validity and representativeness in the field of HRV research. These nine features cover the core dimensions of both time and frequency domains, allowing for the characterization of the dynamic balance of the autonomic nervous system from multiple perspectives (19, 23, 24). Specifically, HR provides the basic physiological background; SDNN reflects the overall regulatory capacity of the autonomic nervous system; rmSSD and pNN50 focus on evaluating parasympathetic nerve activity. In frequency domain analysis, LF and HF allow researchers to distinguish the contributions of sympathetic and parasympathetic nerves; VLF is closely related to metabolic and thermoregulatory processes in long-term monitoring; TSP integrates the overall energy distribution, providing a global input for machine learning models.

**Table 1:** Nine features of the HRV dataset.

Feature	Unit	Biological definition
HR	bpm	Heart rate
SDNN	ms	Standard deviation of NN (normal R-peaks) intervals
rmSSD	ms	Square root of the mean of the sum of the squares of differences between successive NN intervals
PNN50	%	Proportion of the number of NN-interval difference of successive NN intervals which are greater than 50 ms divided by the total number of NN intervals
VLF	ms^2^	Very low frequency power (0.003–0.04 Hz)
LF	ms^2^	Low frequency power (0.04–0.15 Hz)
HF	ms^2^	High frequency power (0.15–0.4 Hz)
TSP	ms^2^	Total spectrum power (<0.4 Hz)
LF/HF	–	LF/HF ratio

The ANS states of all participants are divided into seven classes, such as fatigue and sympathetic nervous system disorder, according to the participants' past medical conditions and the doctors' diagnosis records. The data labeling process employed a combination of the clinical diagnostic data and HRV data analysis (19, 23, 25). HRV data was collected in the hospital, and the participants had already undergone relevant clinical diagnoses, including: head-up tilt test, deep breathing test, valsalva maneuver, and clinical questionnaires. The following physiological and biochemical data were obtained: baseline blood pressure, BMI, age, medical history, cortisol, and catecholamines. Based on these diagnostic and physiological data, combined with HRV data, physicians categorized participants into seven groups. For example: Stress: high cortisol levels or high PSS-10 score; ANS Balance: low COMPASS-31 score, normal blood pressure, and no relevant medical history. These seven classes are used as the labels of the training dataset, as shown in Table 2.

**Table 2:** Seven labels of the HRV dataset.

Label	Autonomic nervous system state	Description
1	Fatigue	Relative hyperactivity of parasympathetic nervous system after long-term stress or energy depletion
2	Sympathetic nervous system disorder	Sympathetic dysfunction, with parasympathetic dominance
3	Autonomic nervous system excitability	The overall activity levels of sympathetic and parasympathetic systems are elevated
4	Autonomic nervous system balance	Sympathetic and parasympathetic nervous are in a reasonable dynamic range
5	Dysautonomia	Autonomic nervous system disorder
6	Stress	Highly excited sympathetic nervous system
7	Vagus nerve disorder	Damage to parasympathetic function

### 2.2 Refined SMOTE-ENN optimization method

The HRV dataset is relatively limited and exhibits an unbalanced distribution across classes. The majority of participants fall into label 4 (ANS balance), comprising 195 cases, while label 2 (sympathetic nervous system disorder) contains only six cases. The r-SMOTE model based on the traditional SMOTE algorithm is proposed to address the extremely imbalanced sample distribution. SMOTE is a technique designed to address class imbalance by generating synthetic minority class samples, which is accomplished by interpolating between existing minority class samples. The first step in the proposed r-SMOTE is feature standardization. The nine HRV features are standardized using z-scores to eliminate discrepancies in dimensionality. Let *z* represent the standardized feature value, and the Equation 1 is obtained:

$$z=\frac{x-\mu }{\sigma }$$ (1)

where *µ* is the mean and *σ* is the standard deviation. This transformation ensures that the interpolation process in the r-SMOTE algorithm is not affected by scale differences in the feature space.

**Figure 2:**
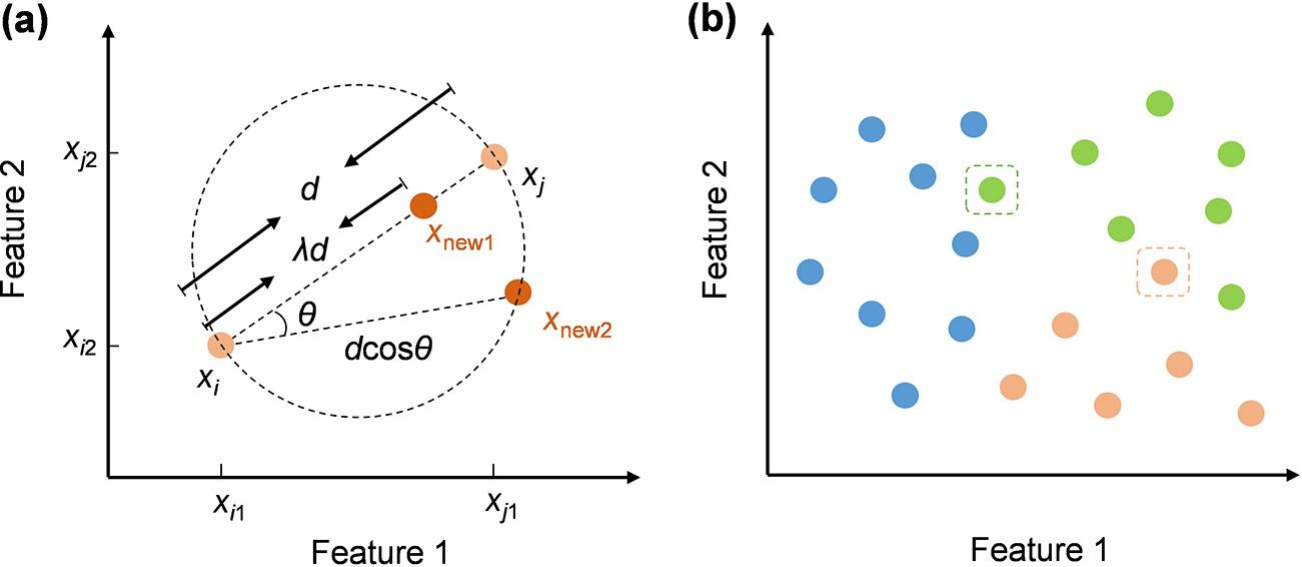
Illustration of the r-SMOTE **(a)** and r-ENN **(b)** principle. **(a)** r-SMOTE generates *x*_new1_ by the in-line linear interpolation and *x*_new2_ by the off-line linear interpolation. **(b)** r-ENN retains a small part of the data on the decision boundary to reduce the risk of overfitting. These data are indicated by the dashed boxes in the figure.

The second step of r-SMOTE involves synthesizing new samples through feature interpolation. For each minority class sample *x*_i_, r-SMOTE calculates its nearest neighbors in the feature space. For example, if the neighbor parameter *k* = 3, r-SMOTE selects three nearest neighbor samples for each minority class sample and generates a new sample that fully utilizes the information from these neighboring samples. Given that the HRV data in the dataset consists of nine features in Table 1, the Euclidean distance between two samples *x*_i_ = (*x*_i1_, *x*_i2_… *x*_i9_) and *x*_j_ = (*x*_j1_, *x*_j2_… *x*_j9_) is defined by Equation 2:

$$d(x_{i},\;x_{j})=\sqrt{\sum_{k=1}^{9}{\left(x_{ik}-x_{\;jk}\right)}^{2}}$$ (2)

where *x*_ik_ and *x*_jk_ are the *k*th feature values of samples *x*_i_ and *x*_j_, respectively. Synthetic samples are generated through in-line and off-line linear interpolation between the minority class sample and its neighboring samples. The synthetic sample *x*_new1_ of linear interpolation is defined by Equation 3:

$$x_{new1}=x_{i}+\lambda (x_{j}-x_{i})$$ (3)

where *λ* is a random value in the range [−1, 1]. This process ensures that the synthetic sample lies on the line crossing the original sample and its neighboring sample, thus maintaining the continuity and correlation of the data. Taking the sample generation of two features as an example, the in-line linear interpolation of r-SMOTE is shown in Figure 2a.

The synthetic sample *x*_new2_ generated by the off-line linear interpolation is located on the circle whose diameter is the line connecting *x*_i_ and *x*_j_, as shown in Figure 2a. The Euclidean distance between *x*_i_ and *x*_new2_ is *d*cos*θ*, where *θ* is a random value in the range [−1/2π, 1/2π]. *θ* = ±1/2π is defined when *x*_new2_ coincides with *x*_i_, and *θ* = 0 when *x*_new2_ coincides with *x*_j._ The coordinate of *x*_new2_ in Figure 2a can be calculated as Equation 4:

$$\begin{array}{rl} & x_{new2,1}=x_{i1}+d\cos\theta \cos\varphi \\ & x_{new2,2}=x_{i2}+d\cos\theta \sin\varphi \\ & \varphi =\arctan\frac{x_{\;j2}-x_{i2}}{x_{\;j1}-x_{i1}}-\theta \end{array}$$ (4)

where *d* is defined by Equation (2). The number of minority samples is effectively increased by in-line and off-line linear interpolation, and the synthetic samples retain the characteristics of the original samples.

Following the r-SMOTE, the r-ENN method is proposed to clean the data based on the traditional ENN (15). r-ENN identifies the *k*-nearest neighbor samples for each sample *x*_i_ in the dataset. If the category of sample *x*_i_ is consistent with the category determined by its *k*-neighbors, the sample is considered correctly classified and retained. A smaller value of *k* ensures that the synthetic samples are closer to the original samples, thus helping to remove noisy data. In this experiment, *k* = 3 is set for r-ENN. Conversely, if the category differs, the sample is considered to lie on the decision boundary and is prone to be misclassified, thus most of these data will be deleted. In contrast to traditional ENN, r-ENN retains a small part of data on the decision boundary to enhance the generalization ability and reduce the risk of overfitting, and these retained boundary data are shown in the dashed box in Figure 2b. In this experiment, 10% to 20% of boundary data are retained.

As a result of applying the refined SMOTE-ENN optimization method, the total number of HRV data samples increased from 321 to 1,172, and the distribution of data across each label is shown in Table 3.

**Table 3:** Original data and refined SMOTE-ENN optimization data.

ANS states	Samples of original data	Samples of refined SMOTE-ENN optimization data
Fatigue	63	142
Sympathetic nervous system disorder	6	209
Autonomic nervous system excitability	9	172
Autonomic nervous system balance	195	125
Dysautonomia	12	175
Stress	24	166
Vagus nerve disorder	12	183

### 2.3 Model construction

20% of the data is randomly selected as the test set, resulting in 937 samples in the training set and 235 samples in the test set. To assess the performance of the refined SMOTE-ENN optimization method across various classification algorithms, four algorithms are chosen for data classification: Support Vector Machine, Random Forest, Neural Network, and K-Nearest Neighbor. These four algorithms encompass discriminative models (SVM), ensemble models (RF), generative and feature learning models (NN), and non-parametric models (KNN), providing a comprehensive assessment of the HRV data. Specifically, SVM maps HRV features to a high-dimensional space using kernel functions, effectively handling non-linear classification problems with small sample sizes (26). RF can automatically capture complex interactions between HRV features by constructing multiple decision trees and introducing randomness (27). Neural networks extract implicit patterns from HRV data that are difficult for traditional statistical methods to capture through multi-layer non-linear transformations (28). KNN performs effective classification based on similarity, and it is very suitable as a baseline model to verify the degree of clustering of HRV data in the feature space since it does not require assumptions about data distribution (29). Other machine learning algorithms do not perform as representatively in HRV classification as these four models. For example, the naive Bayes classifier assumes that all input features are independent; however, there is strong coupling between HRV features. For instance, LF and TSP are highly correlated mathematically and physiologically, and rmSSD and HF both reflect parasympathetic nervous activity. This interdependence violates the basic assumptions of Bayes’ theorem, thus reducing classification accuracy (30).

#### 2.3.1 Support vector machine

The hyperparameters *C*, *γ*, and kernel function need to be optimized for the SVM model. The regularization parameter *C* controls the trade-off between model complexity and training error. The optimization objective of the SVM is determined by Equation 5:

$$\min_{\omega }\frac{1}{2}\|\omega {\|}^{2}+C\sum_{i=1}^{N}\xi_{i}$$ (5)

where *ω* represents the normal vector of the hyperplane, determining the direction of the classification boundary; *C* is the regularization parameter, *N* is the number of training samples; *ξ*_i_ denotes the slack variable, which allows a small number of training samples to be on the incorrect side of the hyperplane. A high value of *C* can lead to overfitting, while a low value may result in underfitting. *C* = 10 is chosen as the regularization parameter through the grid search optimization.

The kernel function determines the shape and properties of the data after mapping to a high-dimensional space. The feature vector of HRV data is complex in this experiment, so the RBF kernel is selected through optimization, which maps the data to an infinite-dimensional space. The RBF kernel is defined by Equation 6:

$$K(x,x^{\prime })=\exp(-\gamma \|x-x^{\prime }{\|}^{2})$$ (6)

where *γ* determines the range of similarity calculations and influences the scope of the RBF kernel function. *γ* = 1 is selected through the grid search optimization in this experiment.

#### 2.3.2 Random forest

Random forest builds multiple decision trees and aggregates the prediction results of each tree to improve the prediction accuracy and generalization ability of the model and reduce the risk of overfitting. This experiment is a classification task, and the voting method is used to determine the final category. Six key hyperparameters are optimized, and the best parameters are: 1,000 trees, *entropy* as the splitting criterion, *sqrt* for maximum features, minimum samples split at 2, minimum samples leaf at 1, and bootstrap sampling set to *False*.

#### 2.3.3 Neural network

**Figure 3:**
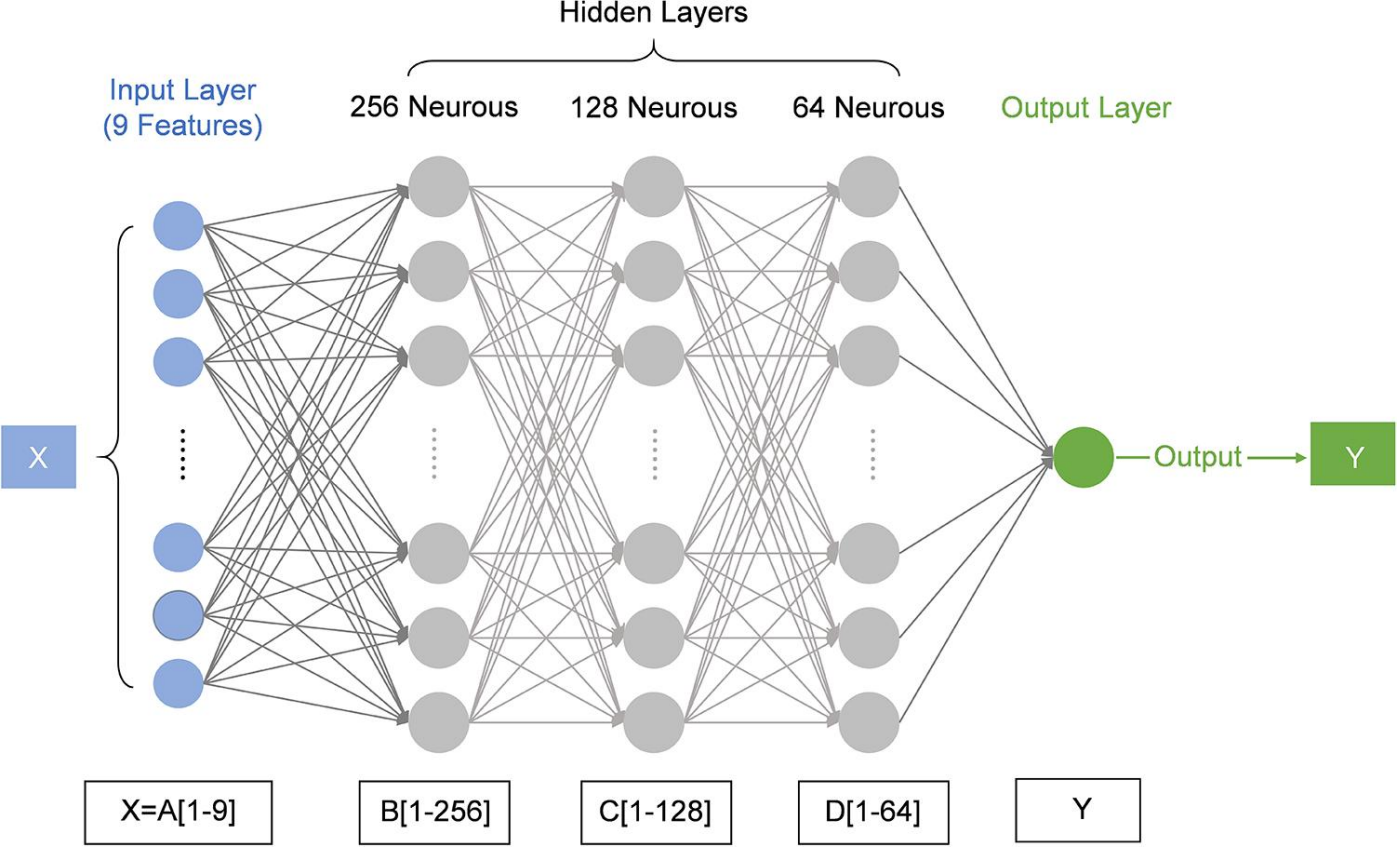
The architecture of the neural network. The network consists of one input layer, three hidden layers, and one output layer. Nine features of the HRV data are input to the network, then transformed by the hidden layers, and finally output in the form of the probability of seven ANS states.

A fully connected neural network is constructed to classify the optimized data, with the network architecture illustrated in Figure 3. The initial fully connected layer maps the 9-dimensional features into a 256-dimensional space. ReLU activation is employed to mitigate the vanishing gradient problem and accelerate training. The He initialization is used to optimize the initial parameters, and the increased feature dimensionality enhances the model's capacity. The output is standardized using Batch Normalization, adjusting the mean and variance of the data to 0 and 1, respectively. To prevent overfitting and enhance the model's generalization ability, the Dropout technique is applied, randomly masking 30% of the neurons. The second fully connected layer performs a nonlinear transformation in the 128-dimensional space, using ReLU activation combined with L2 regularization to prevent overfitting, deepen the network, and improve the feature abstraction ability. Batch Normalization and Dropout are also employed here to further stabilize the output distribution and increase the model's robustness. The feature dimension is then compressed from 128 to 64. Finally, the probability distribution across the seven labels is generated through the Softmax output layer.

#### 2.3.4 K-nearest neighbor

KNN is an instance-based learning algorithm that performs classification by measuring the distance between the input sample and the existing samples in the training dataset. For a given new sample, KNN calculates the distance between this sample and all the samples in the training set, selects the *k* nearest neighbors based on the smallest distances, and predicts the output of the sample according to the category of the neighbors. In this study, the hyperparameters of the KNN classifier are optimized using grid search. The optimal parameters selected are the algorithm = *auto*; distance = *Euclidean*; number of nearest neighbors = 3.

The optimized hyperparameters of SVM, RF, and KNN after grid search are summarized in Table 4. For the NN model, an empirical architecture design is adopted, combined with an early stopping mechanism and an adaptive learning rate adjustment strategy to prevent overfitting, rather than the traditional grid search optimization.

**Table 4:** Hyperparameters with optimization of the four methods.

Methods	Hyperparameters with optimization
SVM	*C* = 10; kernel: RBF; *γ* = 1
RF	1,000 trees; splitting criterion: *entropy*; maximum features: *sqrt*; minimum samples split: 2; minimum samples leaf: 1; bootstrap sampling: *False*
NN	number of hidden layers: 3; neurons in the three hidden layers: 256, 128, 64; activation function: ReLU; Dropout: 0.3
KNN	algorithm: *auto*; distance: *Euclidean*; number of nearest neighbors: 3

## 3 Results and discussion

The overall performance of the four classification models is presented in Table 5. Accuracy is defined as the ratio of the number of samples correctly predicted to the total number of samples, reflecting the overall performance of the model. Precision is defined as True Positive/(True Positive + False Positive), indicating the reliability of the model's prediction of positive samples. Recall is defined as True Positive/(True Positive + False Negative), reflecting the model's ability to capture positive samples. The F1 score is defined as the harmonic mean of the precision and recall, reflecting the balance between these two parameters. The classification accuracy for all models exceeds 0.91, and the F1 score exceeds 0.9. Specifically, KNN demonstrates the best performance, with the overall classification accuracy of 0.97 and F1 score of 0.96, indicating that all four machine learning models have achieved strong classification results on the optimized dataset.

**Table 5:** Classification results of the data using refined SMOTE-ENN optimization.

Method	Accuracy	Precision	Recall	F1 score
SVM	0.93	0.92	0.92	0.92
RF	0.94	0.94	0.93	0.93
NN	0.91	0.91	0.90	0.90
KNN	0.97	0.97	0.96	0.96
Mean	0.94	0.94	0.93	0.93

**Figure 4:**
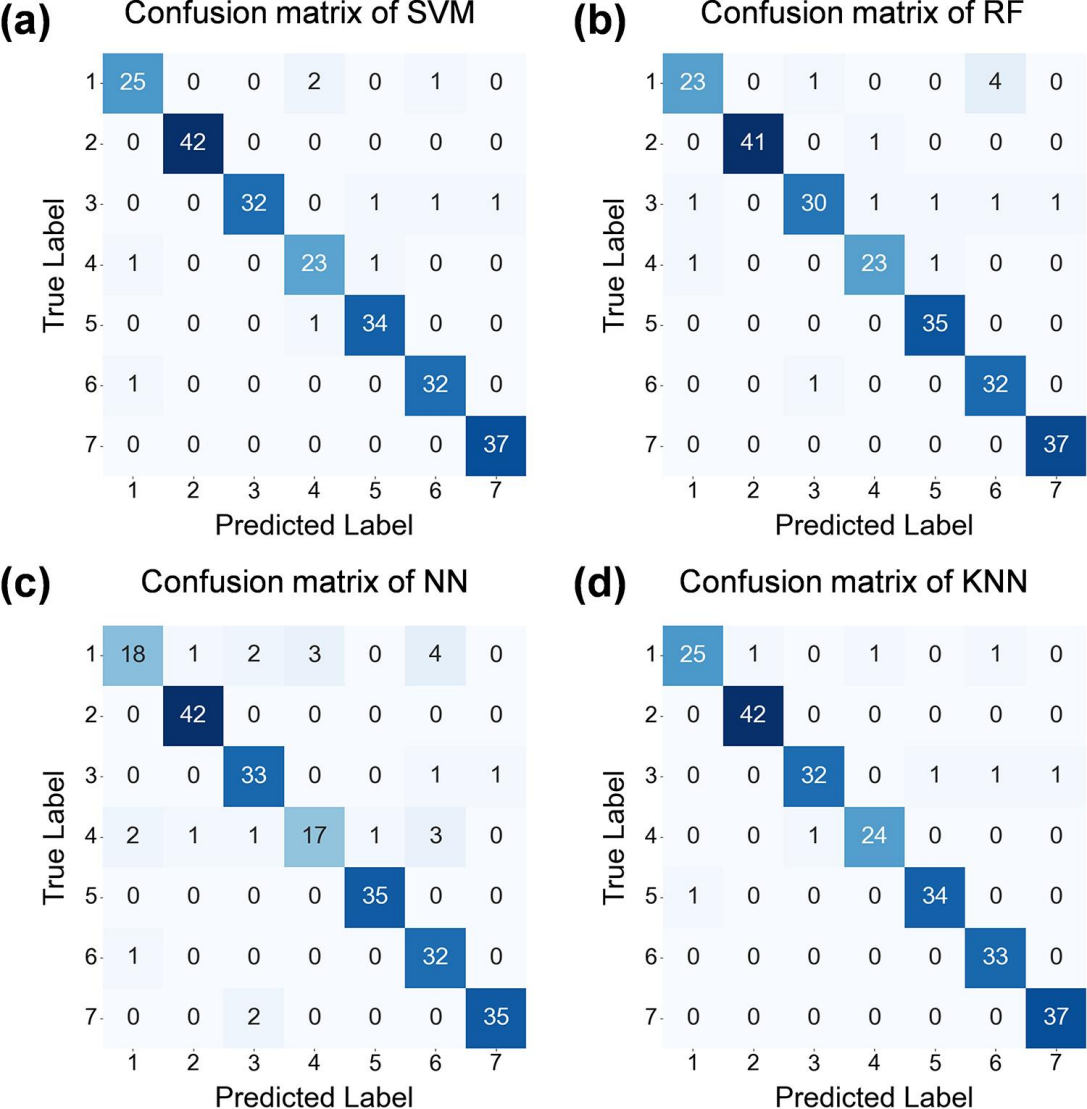
Confusion matrices of the four machine learning models. **(a)** Confusion matrix of SVM. **(b)** Confusion matrix of RF. **(c)** Confusion matrix of NN. **(d)** Confusion matrix of KNN. The classification accuracy for all models exceeds 0.91, and the F1 score exceeds 0.9. KNN demonstrates the best performance among them, with the overall classification accuracy of 0.97 and F1 score of 0.96.

The confusion matrices of the four machine learning models are shown in Figure 4. The classification results for labels 2, 5, 6, and 7 are particularly strong, with label 2 showing slight overfitting in the SVM model. This may be due to the limited amount of original data for label 2, resulting in the synthetic data being overly concentrated in the feature space. To solve this issue, the *k*-value can be appropriately increased in the r-ENN to introduce a small amount of noisy data, thereby enhancing the model's generalization ability. The classification accuracy for label 1 is the lowest across the four models, which may be attributed to the generation of excessive noise data for this label by r-SMOTE. To address this issue, a smaller value of *λ* and *θ* could be used in r-SMOTE to ensure that the synthetic data is closer to the original data in the feature space.

The classification results on the data using the traditional SMOTE method of the four models are summarized in Table 6. For the SMOTE optimization, the mean accuracy, precision, recall, and F1 score are 0.82, 0.82, 0.83, 0.82, respectively. After applying the refined SMOTE-ENN optimization as shown in Table 5, the mean accuracy, precision, recall, and F1 score are 0.94, 0.94, 0.93, 0.93, respectively, improving by 0.12, 0.12, 0.10, and 0.11, respectively. These improvements demonstrate that the refined SMOTE-ENN method is more suitable for optimization on datasets with small sample sizes and imbalanced data distributions compared with the traditional SMOTE, which effectively enhances the classification performance of the four models.

**Table 6:** Classification results of the data using traditional SMOTE optimization.

Method	Accuracy	Precision	Recall	F1 score
SVM	0.8	0.81	0.82	0.81
RF	0.81	0.81	0.82	0.81
NN	0.84	0.83	0.85	0.84
KNN	0.82	0.82	0.81	0.81
Mean	0.82	0.82	0.83	0.82

**Figure 5:**
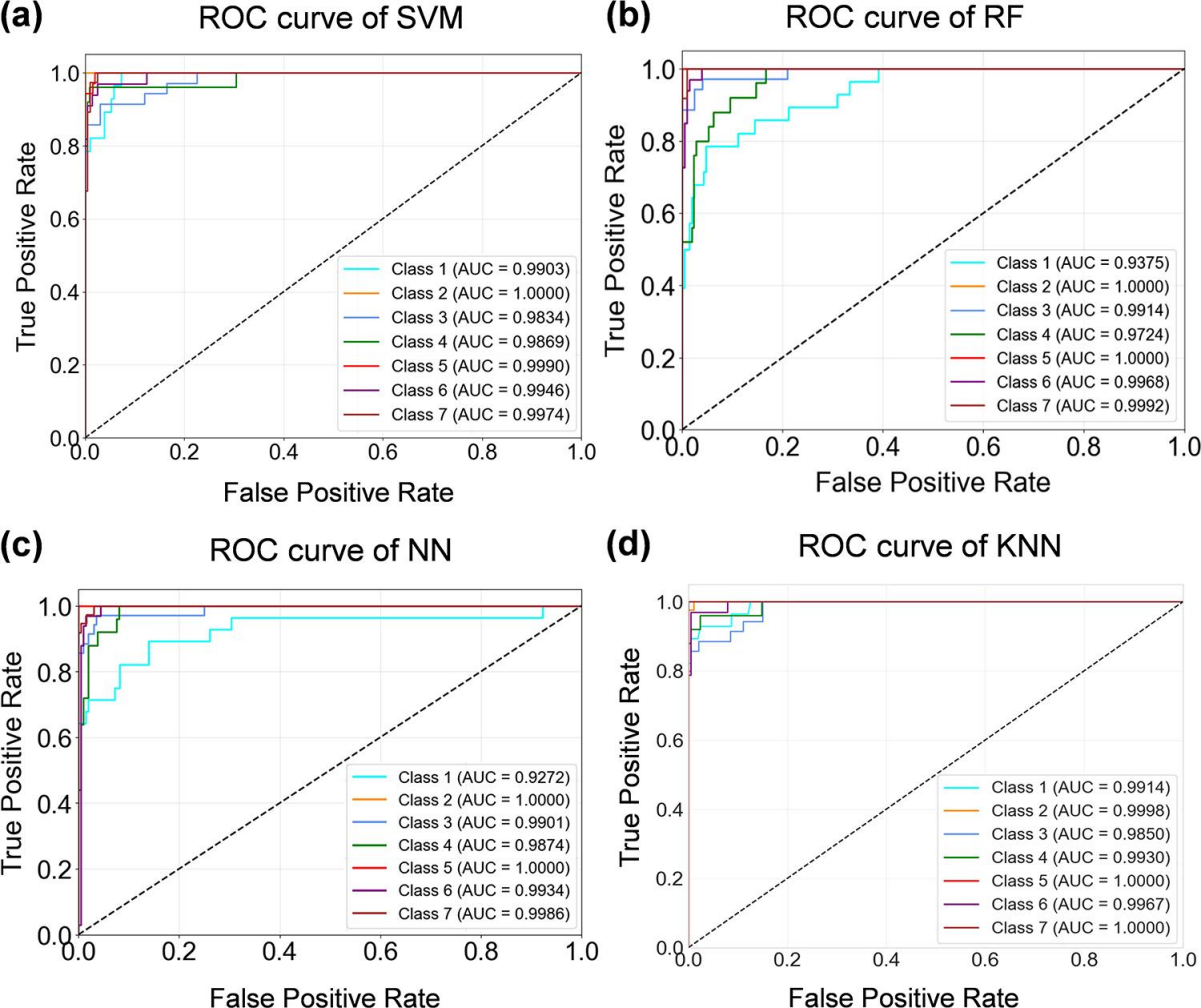
The ROC curves of the four machine learning models. **(a)** ROC curve of SVM. **(b)** ROC curve of RF. **(c)** ROC curve of NN. **(d)** ROC curve of KNN. All curves are positioned close to the upper left corner, and the AUC values exceed 0.92 for all models, demonstrating their capability to effectively distinguish between positive and negative samples.

The Receiver Operating Characteristic (ROC) curves of the four machine learning models are calculated to evaluate the classification performance of the model, as shown in Figure 5. True Positive Rate (TPR) is taken as the vertical axis, which is defined as TPR = True Positive/(True Positive + False Negative), indicating the rate of samples that are correctly predicted as positive and are truly positive. False Positive Rate (FPR) is taken as the horizontal axis, which is defined as FPR = False Positive/(False Positive + True Negative), indicating the rate of samples that are incorrectly predicted as positive and are truly negative. The ROC curves of all models reach a TPR of more than 80% when the FPR is 10%, which is notably higher than the diagonal line, suggesting that the four models exhibit strong discriminative ability. All curves are positioned close to the upper left corner, and the AUC values exceed 0.92 for all models, demonstrating their capability to effectively distinguish between positive and negative samples. Two similar previous studies are compared with this work, and the results are shown in Table 7. Compared to previous classification results using the SMOTE method on large samples, the refined SMOTE-ENN has certain advantages in optimizing and classifying small datasets.

**Table 7:** Comparison results of this work with previous studies.

Study	Data size	Features	Methods	Evaluation results
Hussain et al. (20)	2,000 samples	SDSD, SD, RMSSD, SDANN, Approximate entropy, Sample entropy, Wavelet entropy, Shannon entropy, Wavelet norm entropy, et al.	SMOTE	**KNN**: Accuracy: 80% sensitivity: 84.61% specificity: 77.27% AUC: 88.63% **NB**: Accuracy: 88.57% sensitivity: 84.61% specificity: 90.90% AUC: 92.65%
Reddy et al. (22)	Approximately 1,480,000 samples	heart rate, mean and standard deviation, EDA signal, peak frequency, Power spectral density, respiratory frequency, et al.	SMOTE	**XGBoost:** Accuracy: 88.22% Precision: 85.83% Recall: 89.14% F-score: 87.16% **Logistic Regression:** Accuracy: 91.36% Precision: 90.21% Recall: 92.36% F-score: 91.19%
This work	321 samples	HR SDNN rmSSD PNN50 VLF LF HF TSP LF/HF	Refined SMOTE-ENN	**KNN:** Accuracy: 97% Precision: 97% Recall: 96% F-score: 96% **RF:** Accuracy: 94% Precision: 94% Recall: 93% F-score: 93%

In some cases, such as the SVM model for label 2, the AUC value reaches 1.0, suggesting potential overfitting. The main reasons are as follows: refined SMOTE-ENN generates new samples by interpolating between two real minority class samples. The synthetic samples are highly similar to the original samples, which might lead to data leakage and overfitting. Furthermore, the experimental dataset has a small number of samples, with some classes having fewer than 10 samples. Refined SMOTE-ENN generates a large amount of data based on these few points, causing the features of that class to be extremely densely distributed in certain narrow regions and resulting in overfitting. To address these issues, samples can be generated only at the classification boundaries, instead of across all minority class samples. Additionally, the data processing flow can be further adjusted to prevent data leakage.

**Figure 6:**
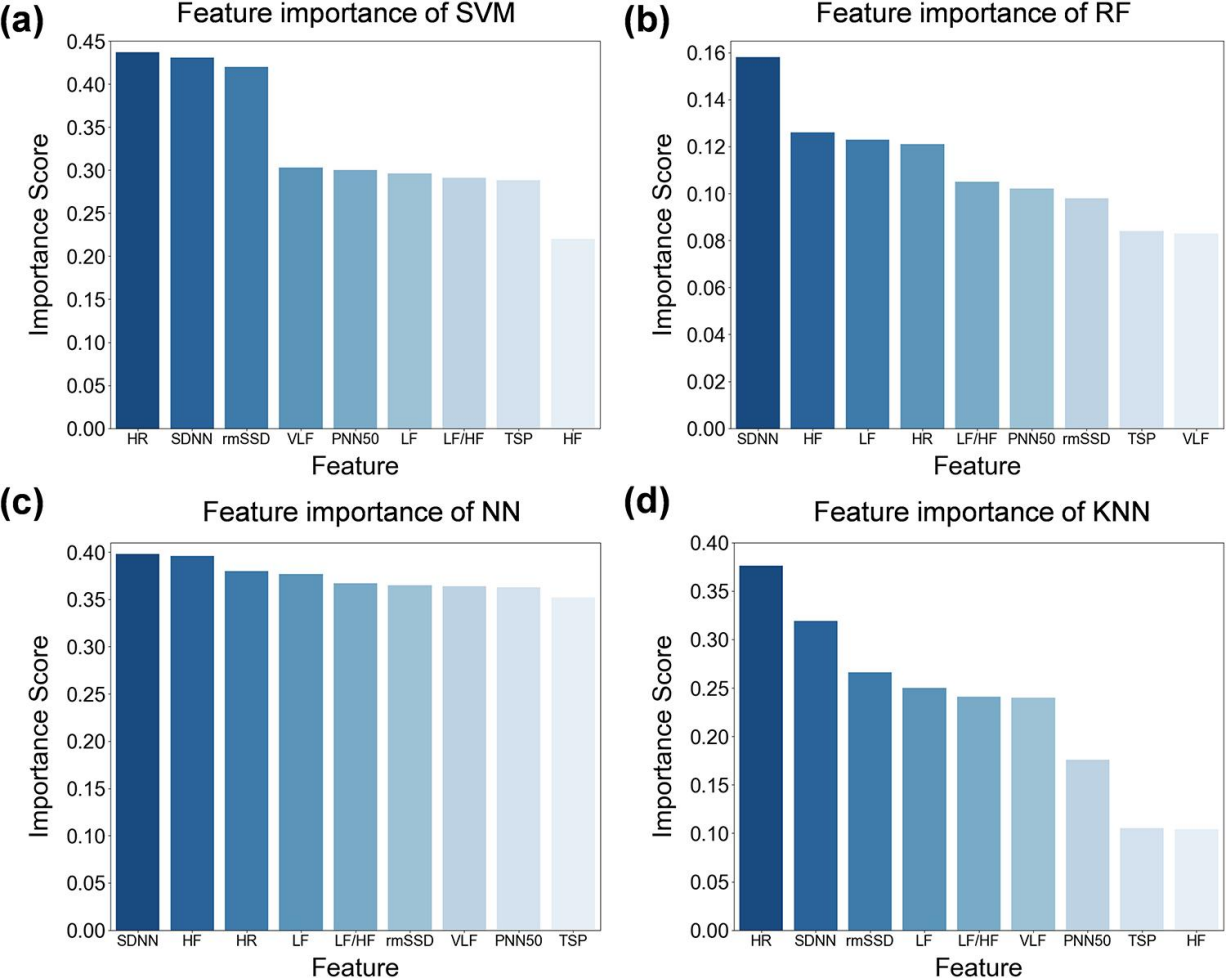
Feature importance distribution of the four machine learning models. **(a)** Feature importance of SVM. **(b)** Feature importance of RF. **(c)** Feature importance of NN. **(d)** Feature importance of KNN. SDNN has the highest mean rank of 1.5, indicating that SDNN is a key indicator of overall heart rate variability and exerts the most significant influence on HRV classification.

The feature importance distribution of the four models is evaluated to examine the clinical interpretability of HRV classification, as shown in Figure 6. The feature importance rankings vary across models. For instance, in the SVM model, the three most influential features are HR, SDNN, and rmSSD, while in the RF model, the top three features are SDNN, HF, and LF. Table 8 presents the ranking of the nine features across all four machine learning models. The results indicate that SDNN has the highest mean rank of 1.5. SDNN represents the standard deviation of all NN intervals, which is a key indicator of overall heart rate variability and exerts the most significant influence on HRV classification. A high SDNN value typically signifies good ANS performance and robust heart adaptation to external stressors. Conversely, changes in the standard deviation of NN intervals are often associated with significant shifts in HRV. The feature with the lowest ranking is TSP, suggesting that TSP has the least impact on HRV classification. This may be due to the fact that, although ECG signals from healthy individuals and depressed patients exhibit varying power levels across different frequency bands, their total power sum is similar, leading to minimal distinction in this feature.

**Table 8:** Feature importance ranking of the four machine learning models.

Method	SDNN	HR	LF	rmSSD	HF	LF/HF	VLF	PNN50	TSP
SVM	2	1	6	3	9	7	4	5	8
RF	1	4	3	7	2	5	9	6	8
NN	1	3	4	6	2	5	7	8	9
KNN	2	1	4	3	9	5	6	7	8
Mean	1.5	2.25	4.25	4.75	5.5	5.5	6.5	6.5	8.25

A limitation of this study is the lack of external validation on public datasets. This is primarily due to the unique nature of the 7-category autonomic nervous system labeling protocol, which does not directly map to the binary or ternary labels (e.g., stress/non-stress) found in currently available public databases. Therefore, the generalization performance of the proposed model on populations with different demographic distributions remains to be verified in future studies. Furthermore, this work is positioned as a pilot study demonstrating the feasibility of identifying complex autonomic states using HRV features. One of the primary goals is to establish the correlation between the proposed 9 features and the 7 functional states within a controlled cohort, and the cross-dataset generalization will be the critical next step of this work.

## 4 Conclusion

Currently, studies on imbalanced data based on SMOTE typically use original datasets with large sample sizes, generally ranging from several thousand to millions. However, its effectiveness in the specific context of depression detection using limited and sparse HRV samples remains to be fully explored. In this work, a refined SMOTE-ENN hybrid optimization method is proposed to address the classification challenges of limited and imbalanced sample distribution. The model utilizes the r-SMOTE algorithm to generate synthetic minority class samples by in-line and off-line linear interpolation, combining it with the r-ENN under-sampling technique to remove noisy data while retaining selected boundary data to reduce the overfitting risk. The experimental results show that after refined SMOTE-ENN optimization, the overall classification accuracy of the four machine learning models exceeds 91%, with the AUC values exceeding 0.92. In comparison to the classification results with classical SMOTE optimization of the four machine learning algorithms, the mean accuracy, precision, recall, and F1 score improved by 0.12, 0.12, 0.10 and 0.11, respectively. The classical SMOTE positions newly generated samples on the lines connecting the original samples in the sample space. The proposed method introduces off-line interpolation, which expands the data space and increases the diversity of generated data through nonlinear transformations introduced by trigonometric functions, overcoming the limitations of linear interpolation in the traditional SMOTE method. The refined SMOTE-ENN optimization method improves the detection performance of machine learning models and provides reliable technical support for the early detection of depression.

Despite the promising results, this study has limitations that point to directions for future research. First, the proposed method effectively addressed the data imbalance, and future studies should focus on collecting larger-scale datasets from multi-center clinical trials to further validate the generalization ability of the model. Second, relying solely on HRV features may limit the feature space; therefore, future work could explore multimodal fusion strategies by integrating HRV with other physiological indicators, such as electroencephalogram signals or voice acoustics. In terms of real-world applications, the proposed method demonstrates certain potential due to its computational efficiency and non-invasive nature, making it suitable for deployment on edge devices, such as smartwatches and health monitoring bands.

## Data Availability

The raw data supporting the conclusions of this article will be made available by the authors, without undue reservation.
